# Improving Treatment and Outcomes for Melioidosis in Children, Northern Cambodia, 2009–2018

**DOI:** 10.3201/eid2704.201683

**Published:** 2021-04

**Authors:** Arjun Chandna, Moritz Bonhoeffer, Thyl Miliya, Keang Suy, Sena Sao, Paul Turner

**Affiliations:** Angkor Hospital for Children, Siem Reap, Cambodia (A. Chandna, M. Bonhoeffer, T. Miliya, K. Suy, S. Sao, P. Turner);; University of Oxford, Oxford, UK (A. Chandna, P. Turner)

**Keywords:** melioidosis, Burkholderia pseudomallei, bacteria, children, pediatrics, treatment, outcomes, trimethoprim/sulfamethoxazole, amoxicillin/clavulanic acid, Cambodia

## Abstract

We report trends in manifestations, treatment, and outcomes of 355 children with culture-confirmed melioidosis over 10 years at a pediatric hospital in northern Cambodia. Bacteremia and presentation with pneumonia were risk factors for death. A total of 39 children recovered after being given only oral antimicrobial drug treatment.

Melioidosis, an infection caused by *Burkholderia pseudomallei*, remains an underrecognized disease, especially in children, in many locations to which it is endemic (*1*,*2*). Diverse clinical manifestations and intrinsic resistance to many antimicrobial drugs used for empirical treatment of sepsis contribute to high mortality rates (*2*–*4*).

Conventionally, antimicrobial drug therapy for melioidosis comprises 2 phases: intravenous treatment for >10 days, followed by a prolonged, oral, eradication phase for a minimum of 12 weeks (*5*,*6*). Localized cutaneous disease might be treatable with oral agents alone, but adherence with eradication therapy is often difficult to achieve (*4*,*5*,*7*–*9*). We report trends in management and outcomes of melioidosis over 10 years at a nongovernmental pediatric hospital in northern Cambodia.

## The Study

This study was approved by the hospital institutional review board (AHC IRB 979-14; 1044-15) and the Oxford Tropical Medicine Research Ethics Committee (OxTREC 550-14). Data on all culture-confirmed case-patients who had *B. pseudomallei* infection during January 1, 2009–December 31, 2018, were collected retrospectively (2009–2013) and prospectively as part of an invasive bacterial infection surveillance study. Retrospective case-patients (the first 173 case-patients) have previously been described and are included to illustrate trends over the decade (*2*).

Retrospective case-patients were identified by searching laboratory logbooks and databases, which were cross-checked against the hospital electronic patient information system. Data were extracted onto a standardized case report form, which was also adapted for contemporaneous capture of prospective case-patients. Repeat searches of the databases were conducted at the end of the study (Appendix Figure 1). The study site, microbiology specimen processing, and case definitions have been described elsewhere (*2*,*8*). We provide the statistical methods used (Appendix). Severe undernutrition was defined as a weight-for-age z-score <–3.

**Figure 1:**
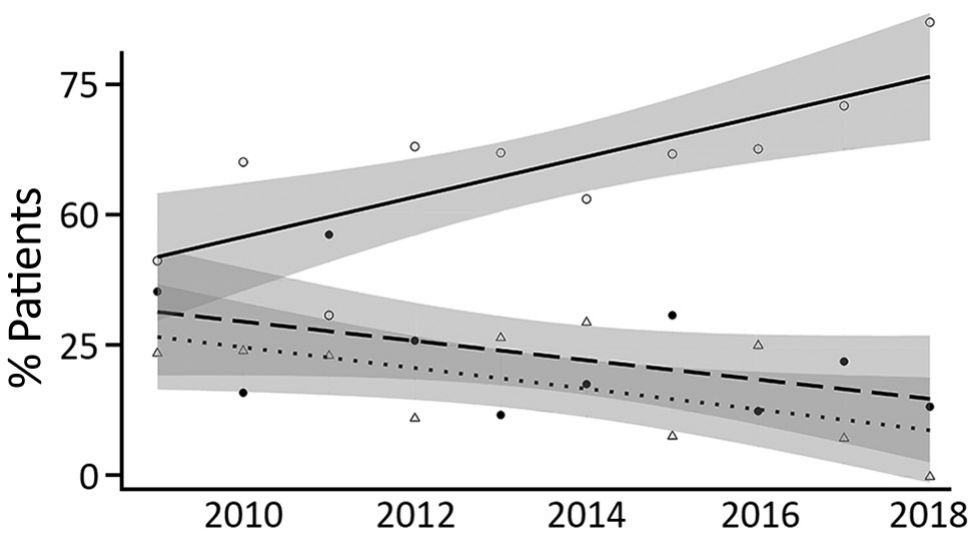
Prevalence of undernutrition for 262 children <10 years of age who had culture-confirmed melioidosis, northern Cambodia, 2009–2018. Linear trend lines indicate nonunderweight children (solid line, open circles: R = 0.76; p = 0.011), children with moderate undernutrition (weight for age z-score [WAZ] <–2) (dashed line, solid circles: R = −0.49; p = 0.150), and children with severe nutrition (WAZ <–3) (dotted line, open triangles: R = −0.59; p = 0.074). Shaded areas indicate 95% CIs for linear trend lines.

Approximately half (57.5%, 255/355) the children with melioidosis were male, and most (82.8%, 294/355) were brought for treatment during the wet season (Appendix Figure 2). Median age was 5.7 years (interquartile range 3.1–9.5 years). Concurrent conditions were infrequent (14/355, 3.9%). Parotitis was the most common manifestation (27.3%, 97/355) (Table 1).

**Table 1:** Characteristics for 355 children who had culture-confirmed melioidosis, northern Cambodia, 2009–2018*

Characteristic	Value
Median age, y (IQR)	5.7 (3.1–9.5)
Sex	
M	255 (57.5)
F	100 (42.5)
Concurrent condition, n = 355	14 (3.9)
Thalassemia	4
Systemic lupus erythematosus	2
Suspected underlying immunodeficiency	2
Asthma	1
Epilepsy	1
Acute lymphoblastic leukemia	1
Congenital heart disease	1
Chronic kidney disease	1
Pure red cell aplasia	1
Clinical manifestations, n = 355	
Parotitis	97 (27.3)
Skin or soft tissue infection	96 (27.0)
Pneumonia	69 (19.4)
Lymphadenitis	58 (16.3)
Meningitis	1 (0.3)
Multifocal infection	12 (3.4)
Other†	8 (2.3)
Unknown‡	15 (4.5)
Management strategy, n = 355	
Admitted case-patients	212 (59.7)
Case-patients admitted at first presentation	145 (40.8)
Empiric treatment with effective intensive-phase therapy	51
Treatment with effective intensive-phase therapy within 48 h	38
Treatment with effective intensive-phase therapy after 48 h	40
No effective intensive-phase therapy received§	11
Treatment information not available	5
Admitted to intensive care unit, n = 212	52 (24.5)
Surviving patients completing 12 weeks of eradication therapy, n = 306	102 (33.3)
No. patients treated successfully with only oral antimicrobial drugs	39

*Values are no. (%) except as indicated. IQR, interquartile range. †Clinical manifestations for patients classified as Other included mandibular osteomyelitis (2), diarrheal disease (2), vaginitis (2), mastoiditis (1), and septic arthritis (1).  ‡Clinical manifestations were unknown for 15 patients: 10 were bacteremic and 5 had *Burkholderia pseudomallei* isolated from pus swabs. §A total of 9 children died within 24 h (before culture results were available), and 2 were switched directly to oral treatment.

Hospital guidelines (introduced in 2012) recommend obtaining blood, throat swab, and urine specimen for culture for all patients who have suspected melioidosis. However, blood was collected for culture for only 157 (44.2%) of 355 case-patients, a throat swab specimen for 31 (8.7%) of 355, and a urine sample for 16 (4.5%) of 355. Use of microbiological testing improved over time (Appendix Table 5, Figure 3). Of those who had blood cultured, 46.5% (73/157) were bacteremic. The proportion of bacteremic children remained consistent over the study period (Appendix Figure 4). For 12 children who were evaluated during 2017–2018, the only positive microbiological specimen was a throat swab specimen.

Treatment data were available for 344 (96.9%) of 355 children. Of these, 140 were admitted when care was initially sought; 89 (63.6%) received an intravenous antimicrobial drug (ceftazidime, meropenem, or imipenem) that had activity against *B. pseudomallei* within 48 hours (Table 1). Eleven children did not receive an effective intravenous drug; 9 died within 24 hours (before culture results were available), and 2 were switched directly to an oral treatment. The time to effective antimicrobial drug therapy did not change over the study period.

The in-hospital case-fatality rate (CFR) was 11.5% (41/355). Median time to death was 2.5 days (interquartile range 1–8 days). Two deaths occurred after discharge for children who had completed 14 days of intensive therapy.

Pneumonia, female sex, and age <5 years were risk factors for death. Among children who had a blood culture, bacteremia was strongly associated with death, as was severe undernutrition in children <10 years of age. Adjusted analyses, including only children <10 years of age who had a blood culture (n = 128), confirmed that bacteremia (odds ratio 57.09, 95% CI 10.80–1,063.54; p<0.001) and pneumonia (odds ratio 3.95, 95% CI 1.22–14.43; p = 0.027) were independently associated with death (Table 2). The annual CFR remained stable, although a nonsignificant decrease was observed for bacteremic children (Appendix Figure 5). This decrease occurred in the context of major reductions in the prevalence of undernutrition (Figure 1).

**Table 2:** Risk factors for death of children who had culture-confirmed *Burkholderia pseudomallei* infection, northern Cambodia, 2009–2018*

Characteristic	Survivors	Nonsurvivors	Unadjusted OR (95% CI); p value	Adjusted OR (95% CI); p value
Whole population, n = 355	n = 312	n = 43		
Female sex	126/312 (40.4)	25/43 (58.1)	2.05 (1.07–3.91); 0.03	1.58 (0.71–3.53); 0.26
Age <5 y	126/312 (40.4)	25/43 (58.1)	2.05 (1.07–3.91); 0.03	0.69 (0.28–1.60); 0.39
Pneumonia†	34/312, (10.9)	35/43 (81.4)	35.77 (15.34–83.41); <0.001	38.99 (16.46–104.01); <0.001
Bacteremia‡	31/114 (27.2)	42/43 (97.7)	112.45 (14.83–852.47); <0.001	70.24 (13.73–1,289.14); <0.001
Severe undernutrition§	32/225 (14.2)	12/37 (32.4)	2.90 (1.32–6.34); 0.008	1.36 (0.51–3.52); 0.53
Children <10 y of age who had blood culture, n = 128	n = 91	n = 37		
Female sex	39/91 (42.9)	20/37 (54.1)	1.57 (0.73–3.38); 0.25	1.24 (0.44–3.46); 0.679
Age <5 y	54/91 (59.3)	23/37 (62.2)	1.13 (0.51–2.47); 0.77	0.70 (0.21–2.18); 0.542
Pneumonia†	31/91 (34.1)	30/37 (81.1)	8.29 (3.27–21.02); <0.001	3.97 (1.22–14.43); 0.027
Bacteremia	29/91 (31.9)	36/37 (97.3)	76.97 (10.05–589.16); <0.001	57.09 (10.80–1,063.54); <0.001
Severe undernutrition	17/91 (18.7)	12/37 (32.4)	2.09 (0.88–4.97); 0.09	2.08 (0.62–7.72); 0.247

*Values are no. positive/no. tested (%) except as indicated. OR, odds ratio. †Pneumonia was defined according to the working diagnosis of the treating clinical team, taking into consideration clinical, laboratory, and radiologic information. A total of 89.9% (62/69) of children who were given a diagnosis of pneumonia had a chest radiograph. ‡Risk for bacteremia assessed in children who had a blood culture collected (n = 157).  §Risk for severe undernutrition (weight-for-age z score <–3) assessed in children <10 y of age (n = 262; a weight measurement was available for 95.6% [262/274] of children <10 y of age). Multivariate analyses adjusted for sex, age <5 years, and pneumonia. Subgroup analysis (n = 128). Includes only children <10 y of age who had a blood culture collected.

**Figure 2:**
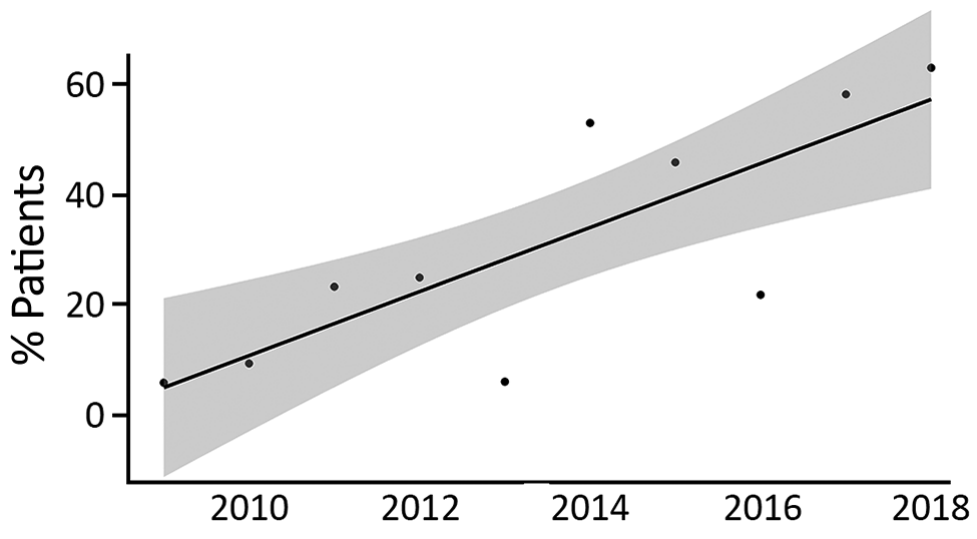
Proportion of 306 surviving children who had culture-confirmed melioidosis and completed >12 weeks of eradication therapy, northern Cambodia, 2009–2018. Shaded area indicates 95% CIs for the linear trend line (R = 0.8; p = 0.006).

Of 312 surviving case-patients, postdischarge information was available for 306 (98.1%), of whom 102 (33.3%) completed >12 weeks of eradication therapy. The proportion of children completing eradication therapy increased substantially during the study period (Figure 2), and trimethoprim/sulfamethoxazole was increasingly likely to be selected as the eradication agent of choice (Appendix Figure 6). Thirty-nine children recovered after receiving only oral antimicrobial drug treatment, including 17 who had lymphadenitis, 14 who had with localized cutaneous disease, and 4 who had parotitis. No culture-confirmed relapses have been reported.

## Conclusions

Our study illustrates the challenges associated with providing care for children who have melioidosis at a pediatric hospital in northern Cambodia. Unlike many hospitals in the region, there was access to level 3 care in a pediatric intensive care unit and an on-site diagnostic microbiology laboratory supported by an active clinical microbiology liaison service (*10*). Nevertheless, 2/10 children admitted because they had melioidosis did not survive to leave the hospital, and only one third of those discharged alive were confirmed as having completed eradication therapy.

Few easily identifiable features exist to alert clinicians to a possible diagnosis of melioidosis. Given the necessity of early and appropriate antimicrobial drug therapy and the intrinsic resistance of *B. pseudomallei* to many first-line antimicrobial drugs, clinicians have the difficult task of maintaining a high index of suspicion while balancing the need for effective antimicrobial stewardship.

Our study might have underestimated the burden associated with melioidosis due to suboptimal diagnostic testing, particularly at the beginning of the study period. The hospital’s clinical microbiology liaison service has expanded over time and now includes 3 weekly infection rounds, invasive bacterial and hospital-acquired infection surveillance, and antimicrobial drug and diagnostic stewardship training as part of the hospital induction program. During 2015, the antimicrobial treatment guidelines became accessible by a mobile application (*10*). In the final 2 years of the study period, 12 children received a confirmed diagnosis only because the clinical team sent a throat swab specimen specifically for *B. pseudomallei* culture.

We observed a slight decrease in the CFR for bacteremic children. Although not significant, this trend cannot be explained by changes in case-mix severity (proportion of bacteremic children) or time to effective antimicrobial therapy. However, it did occur in the context of substantial gains in the nutritional status of the population. Malnutrition might potentiate severity of melioidosis in children and is a well-recognized risk factor in other pediatric infections (*4*,*11*).

The fact that only one third of children surviving to hospital discharge went on to successfully complete 12 weeks of eradication therapy illustrates both the challenge associated with ambulatory care in resource-constrained settings and the gains that can be achieved over time. During 2009, only 5% of case-patients completed 12 weeks of eradication therapy, compared with 52% during 2018. Furthermore, by the end of the study period, amoxicillin/clavulanic acid had been replaced by trimethoprim/sulfamethoxazole acid as the eradication drug of choice, consistent with current recommendations (*5*,*6*).

This study lends support to the idea that certain *B. pseudomallei* infections might be treatable with oral agents alone (*5*). The 39 cases described herein add to >26 previously reported case-patients for whom successful outcomes have been achieved in the absence of parenteral therapy (*4*,*8*,*9*).

In summary, melioidosis remains a major disease in children in Cambodia. Proactive microbiological specimen collection is critical to confirming the diagnosis. Although adherence with prolonged eradication therapy is challenging, over time, improvements can be realized. Adherence will become increasingly essential if outpatient oral treatment regimens are to be considered in resource-limited settings, as they are in some high-income, disease-endemic locations (*4*).

Appendix Additional information on improving treatment and outcomes for melioidosis in children, northern Cambodia, 2009–2018.
