# Supplementary material for: Improving Treatment and Outcomes for Melioidosis in Children, Northern Cambodia, 2009–2018
Source: Emerg Infect Dis. 2021 Apr;27(4):1169–72. doi: 10.3201/eid2704.201683 (PMC8007301; doi:10.3201/eid2704.201683)
Supplement: Appendix — Additional information on improving treatment and outcomes for melioidosis in children, northern Cambodia, 2009–2018. [file 20-1683-Techapp-s1.pdf]

Article DOI: https://doi.org/10.3201/eid2704.201683

# Improving Treatment and Outcomes for Melioidosis in Children, Northern Cambodia, 2009–2018

## Appendix

### Statistical Methods

Data were analyzed by using R software version 3.6.1 (https://www.r-project.org). For children <10 years of age, weight-for-age z-scores were calculated according to World Health Organization Child Growth Standards (https://www.who.int/toolkits/child-growth-standards) and the “anthro” package in R. Descriptive statistics were used to describe relevant characteristics of the cohort. Medians and interquartile ranges were used to report non-normally distributed variables (age and time to death).

Variables for univariable analysis were selected according to clinical relevance and biologic plausibility (sex, age <5 years, clinical diagnosis of pneumonia, severe undernutrition, and presence of bacteremia) to determine their association with death. Unadjusted odds ratios and 95% CIs were calculated. The denominator for analysis of sex, age, and clinical presentation was the entire study population (n = 355). The analysis for bacteremia was restricted to the number of children that had a blood culture collected (n = 157) and for undernutrition to children <10 years of age (n = 274) because the World Health Organization Child Growth Standards are only validated up until this age. An α of 0.05 was defined as being statistically significant.

For the purposes of these analyses, the 12 children (12/274, 4.4%) <10 years of age for whom a weight measurement was not available were excluded. The 15 case-patients for whom clinical presentation was unknown were classified as not having presented with pneumonia: 10 of these 15 case-patients were bacteremic and assumed to have presented with undifferentiated sepsis, and the remaining 5 had *Burkholderia pseudomallei* isolated from pus swabs and were therefore believed unlikely to have had a pneumonic presentation.

Multivariable logistic regression was used to identify any variables that were independently associated with death. All variables that were biologically plausible (i.e., all

variables selected for univariable analyses) were included in the multivariable analysis. Variables were not formally assessed for collinearity or interactions. Analysis of residuals confirmed the assumptions of linearity. Adjusted odds ratios and 95% CIs were calculated. Regression equations, coefficients of each explanatory variable, and goodness-of-fit of models assessed by the Hosmer and Lemeshow test ($\chi^2$) are indicated (Appendix Tables 1–5).

Correlation analyses were used to determine the trend over time for explanatory and outcome variables (explanatory: undernutrition and bacteremia; outcome: in-hospital case fatality rates, blood culture collection rate, choice of antimicrobial agent for eradication therapy, and successful completion of eradication therapy). The value of the Pearson correlation coefficient (R) was reported. An $\alpha$ of 0.05 was defined as being statistically significant.

**Appendix Table 1.** Multivariable logistic regression model to identify risk factors for death for children who had culture-confirmed *Burkholderia pseudomallei* infection (n = 355), northern Cambodia, 2009-2018*

| Variable | Coefficient (95% CI) | p value |
|---|---|---|
| Intercept | −0.11 (−0.76 to –0.52) | 0.73 |
| Age<5 years | −0.38 (−1.26 to –0.47) | 0.39 |
| Pneumonia | 3.66 (2.80–4.64) | <0.001 |
| Female sex | 0.45 (−0.34 to 1.26) | 0.72 |

*Mortality rate = −0.11 + (−0.38 x age <5 years) + (3.66 x pneumonia) + (0.45 x female). Hosmer and Lemeshow goodness of fit: $\chi^2$ = 1.19; p = 1.00.

**Appendix Table 2.** Multivariable logistic regression model to identify risk factors for death for children who had culture-confirmed *Burkholderia pseudomallei* infection and had a blood culture collected (n = 157), northern Cambodia, 2009-2018*

| Variable | Coefficient (95% CI) | p value |
|---|---|---|
| Intercept | −0.77 (−1.60 to 0.00) | 0.06 |
| Bacteremia | 4.25 (2.62–7.16) | <0.001 |
| Age <5 years | −0.57 (−1.75 to –0.49) | 0.30 |
| Pneumonia | 1.73 (0.62–2.97) | 0.003 |
| Female sex | 0.31 (0.67–1.29) | 0.53 |

*Mortality rate = −0.77 + (−0.57 x age <5 years) + (1.73 x pneumonia) + (0.31 x female). Hosmer and Lemeshow goodness of fit: $\chi^2$ = 7.77; p = 0.46.

**Appendix Table 3.** Multivariable logistic regression model to identify risk factors for death for children who had culture-confirmed *Burkholderia pseudomallei* infection and were <10 years of age (n = 262), northern Cambodia, 2009-2018*

| Variable | Coefficient (95% CI) | p value |
|---|---|---|
| Intercept | −1.10 (−1.06 to –0.85) | 0.82 |
| Age <5 years | −0.38 (−1.32 to –0.53) | 0.41 |
| Pneumonia | 3.23 (2.32–4.27) | <0.001 |
| Severe acute malnutrition | 0.30 (−0.68 to 1.26) | 0.53 |
| Female sex | 0.23 (−0.63 to 1.08) | 0.60 |

*Morality rate = −1.10 + (−0.38 x age <5 years) + (3.23 x pneumonia) + (0.30 x severe acute malnutrition) + (0.23 x female). Hosmer and Lemeshow goodness of fit: $\chi^2$ = 0.53; p = 1.00.

**Appendix Table 4.** Multivariable logistic regression model to identify risk factors for death for children who had culture-confirmed *Burkholderia pseudomallei* infection, had a blood culture collected and were <10 years of age (n = 128), northern Cambodia, 2009-2018*

| Variable | Coefficient (95% CI) | p value |
|---|---|---|
| Intercept | −1.19 (−2.57 to –0.04) | 0.07 |
| Bacteremia | 4.04 (2.38–6.97) | <0.001 |
| Age <5 years | −0.36 (−1.58 to –0.78) | 0.54 |
| Pneumonia | 1.38 (0.20–2.67) | 0.03 |
| Severe acute malnutrition | 0.73 (−0.48 to 2.04) | 0.25 |
| Female sex | 0.21 (−0.82 to 1.24) | 0.68 |

*Mortality rate = −1.19 + (4.04 x bacteremia) + (−0.36 x age <5 y) + (1.38 x pneumonia) + (0.73 x severe acute malnutrition) + (0.21 x female). Hosmer and Lemeshow goodness of fit: $\chi^2$ = 7.03; p = 0.53.

**Appendix Table 5.** Children who provided a throat swab specimen or urine sample for *Burkholderia pseudomallei* culture by year (n = 355), northern Cambodia, 2009–2018

| Sample | 2009 | 2010 | 2011 | 2012 | 2013 | 2014 | 2015 | 2016 | 2017 | 2018 |
|---|---|---|---|---|---|---|---|---|---|---|
| Throat swab specimen | 1 | 1 | 1 | 0 | 0 | 0 | 1 | 2 | 15 | 10 |
| Urine culture | 0 | 0 | 0 | 0 | 0 | 0 | 0 | 1 | 5 | 10 |

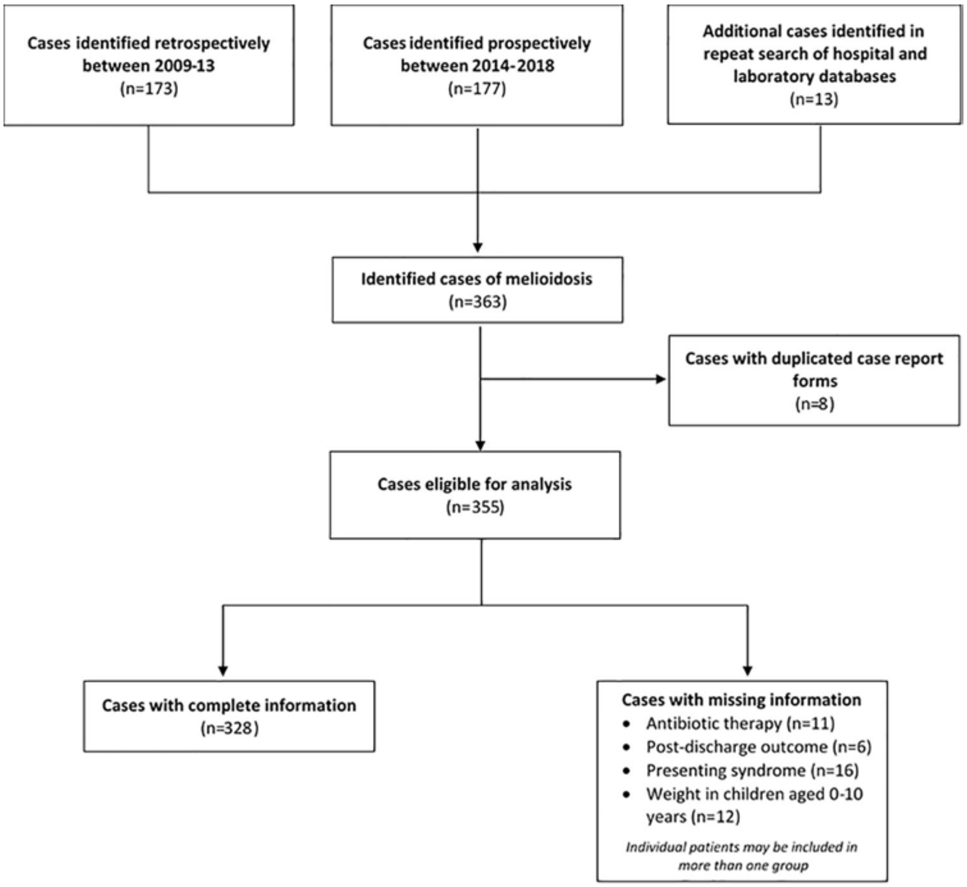


**Appendix Figure 1.** Identification of children who had *Burkholderia pseudomallei* infection, northern Cambodia, 2009–2018.

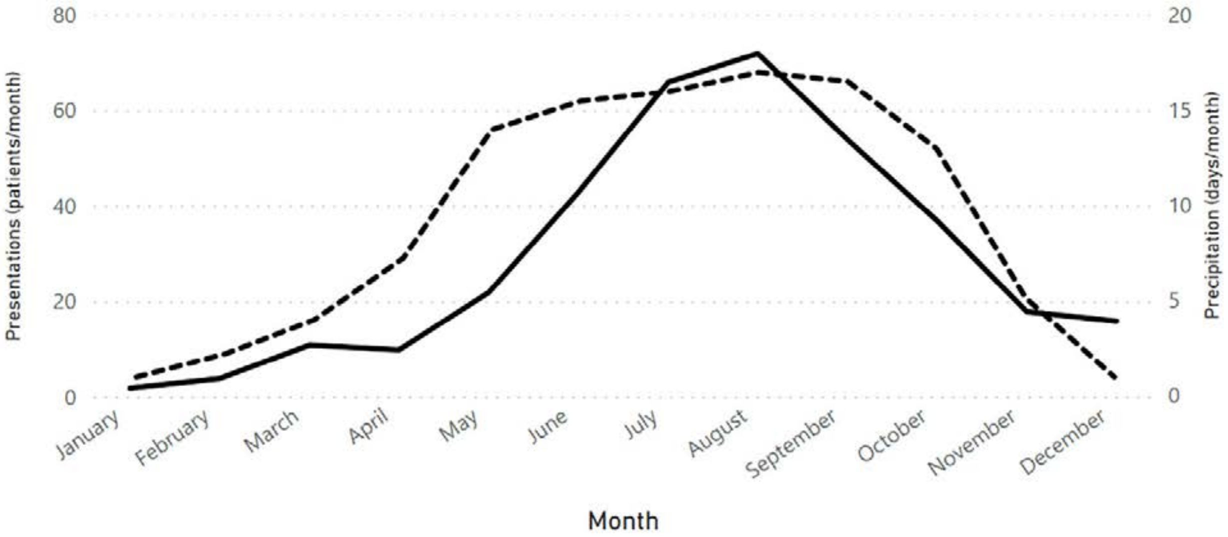


**Appendix Figure 2.** Seasonality for presentation of children with culture-confirmed melioidosis, northern Cambodia, 2009–2018. Solid line indicates no. cases per calendar month. Dashed line indicates average number of days of rain per calendar month (climate data were obtained from https://en.tutiempo.net/climate/ws-489660.html).

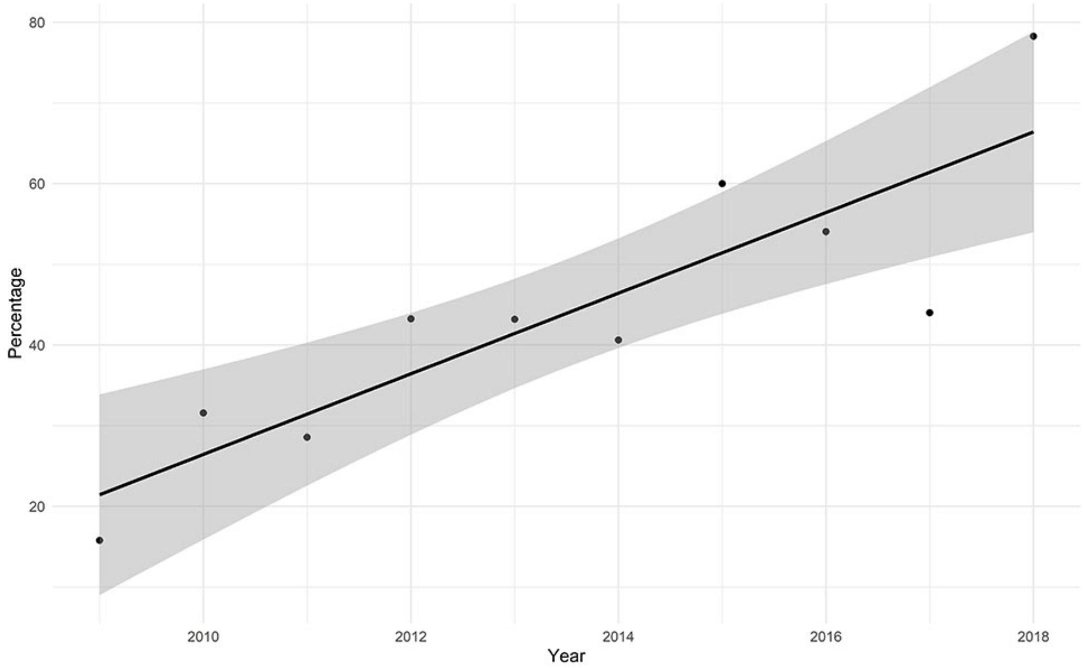


**Appendix Figure 3.** Trend in proportion of children who had melioidosis and for whom blood was collected for *Burkholderia pseudomallei* culture (n = 355), northern Cambodia, 2009–2018. Blue indicates 95% CI of linear trend line (R = 0.870; p = 0.001). R, Pearson coefficient reported for correlation.

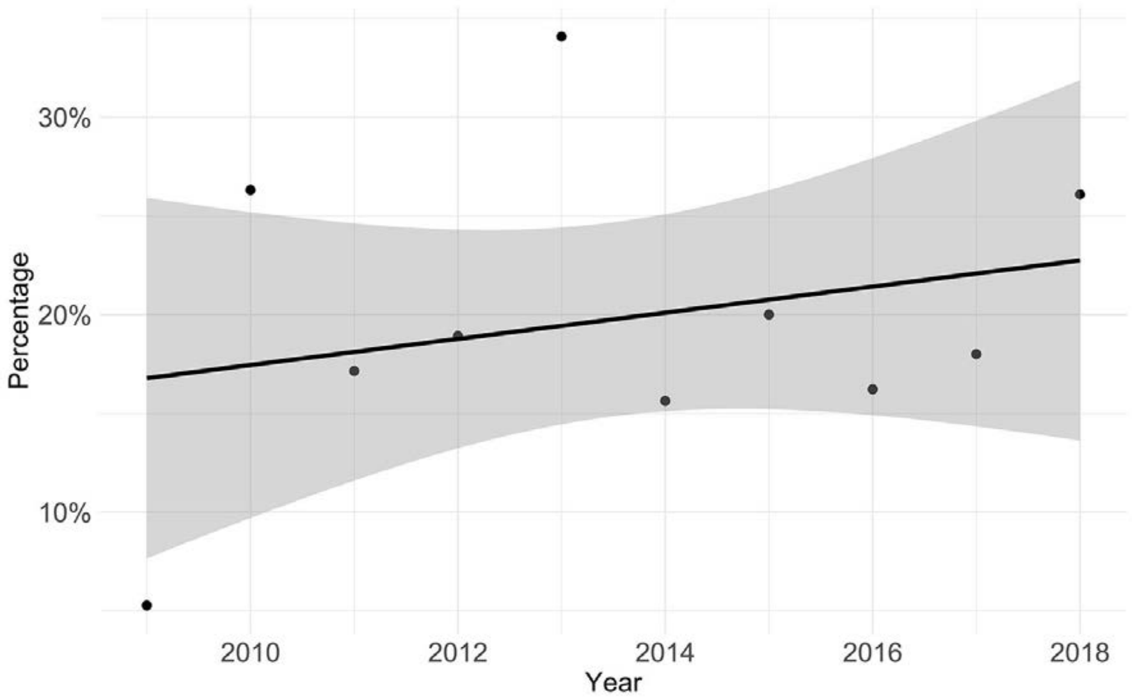


**Appendix Figure 4.** Trend in proportion of children with *Burkholderia pseudomallei* isolated from blood cultures (n = 355) for children who had melioidosis, northern Cambodia, 2009–2018. Blue indicates 95% CI of linear trend line (R = 0.260, p = 0.47). R, Pearson coefficient for correlation.

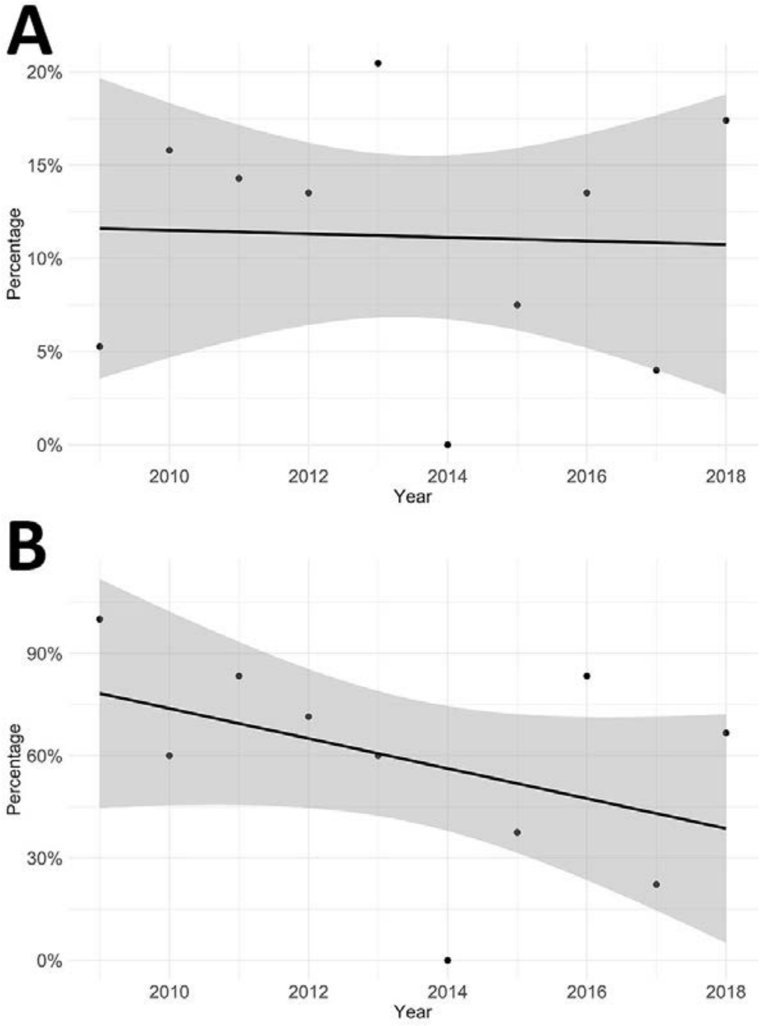


**Appendix Figure 5.** A) Annual in-hospital case-fatality rate for 355 children with melioidosis, northern Cambodia, 2009–2018. Blue indicates 95% CI of linear trend line (R = −0.044; p = 0.9). R, Pearson coefficient for correlation. B) Annual in-hospital case-fatality rate for children with *Burkholderia pseudomallei* isolated from blood culture (n = 73).Blue indicates 95% CI confidence interval of linear trend line (R = −0.44;  p = 0.21). R, Pearson coefficient for correlation.

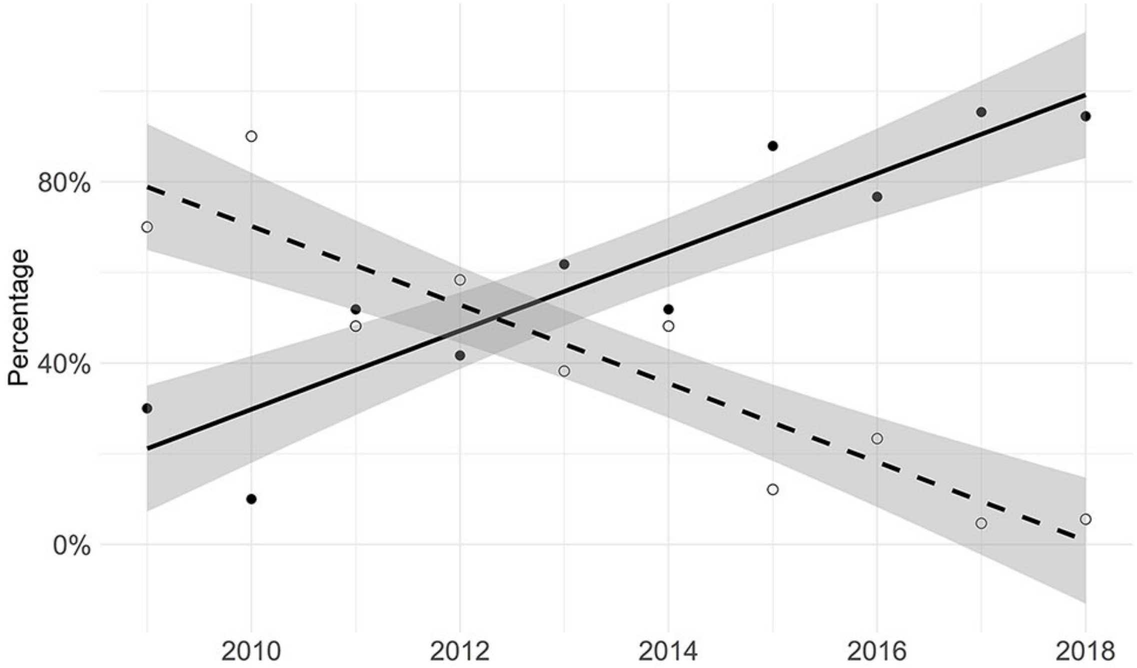


**Appendix Figure 6.** Change in selection of oral antimicrobial drug for eradication therapy for 276 children who had melioidosis and were given either trimethoprim/sulfamethoxazole or amoxicillin/clavulanic acid, northern Cambodia, 2009–2018. Solid line and solid circles (R = 0.92; p<0.001) indicate trimethoprim/sulfamethoxazole, and dashed line and open circles indicate amoxicillin/clavulanic acid. Blue area indicate 95% CIs of linear trend lines. R, Pearson coefficient for correlation.
